# Effect of linoleic acid supplementation on in vitro maturation, embryo development and apoptotic related gene expression in ovine

**Published:** 2016-04

**Authors:** Ebrahim Amini, Reza Asadpour, Leila Roshangar, Razi Jafari-Joozani

**Affiliations:** 1 *Department of Clinical Sciences, Faculty of Veterinary Medicine, University of Tabriz, Tabriz, Iran.*; 2 *Department of Anatomical Sciences, Faculty of Medicine, Tabriz University of Medical Sciences, Tabriz, Iran. *

**Keywords:** *Linoleic acid*, *In vitro oocyte maturation techniques*, *Embryo culture techniques*, *Gene expression*

## Abstract

**Background:**

Linoleic acid (LA) is a polyunsaturated fatty acid present in high concentrations in follicular fluid, when added to maturation culture media, it affects oocyte competence.

**Objective:**

In the present study, we investigated effect of linoleic acid supplementation on in vitro maturation, embryo development and apoptotic related gene expression in ovine

**Materials and Methods:**

The experiments conducted on 450 ovine Cumulus-oocyte complexes (COCs) with homogenous ooplasm and more than two compact layers of cumulus cells. For in vitro maturation COCs were randomly allocated into four treatment groups for 24 hr period. Treatment groups were as follow: control maturation media, 0 µM LA, 50 µM LA, 100 µM LA and 200 µM LA. The cumulus cell expansion and blastocysts rates were recorded. Total RNA was isolated from embryo pools, reverse transcribed into cDNA, and subjected to apoptotic gene expression by real-time PCR.

**Results:**

Highest concentration (200 µM/mL) of LA significantly decreased the rate of fully expanded cumulus cells 24 hr after in vitro maturation (IVM) and the percentage of blastocyste rate compared with the control (p<0.05). These inhibitory effects were associated with an increased in relative mRNA expression of Bax (Bcl-2- associated X) gene compared with controls.

**Conclusion:**

Data obtained in present study suggest that low concentration of LA used for maturation had no deleterious effect on subsequent embryonic development compared to high concentration of LA. Relative expression of Bcl-2 (B-cell lymphoma 2) and Bax in embryos seems to be associated with LA concentration.

## Introduction

Supplementation of diet with polyunsaturated fatty acids (PUFAs) can influence the reproductive performance animal (1). PUFAs including omega-6, omega-3, and essential fatty acids (FAs) which are important for health but cannot be synthesized in body and must be provided by diet (2). They are well known to alter membrane lipid composition, cellular metabolism, cholesterol metabolism, regulation of steroidogenesis, signal transduction and gene expression regulation in various tissues (3, 4). The animals fed with FAs have altered proportions of FAs in follicular fluid and show improved reproductive functions in bovine and ovine (5-8). The effect of adding FAs to oocyte maturation culture media on oocyte competence has also been studied, outcome depends on type and concentration of FA to which oocytes is exposed (9).

Linoleic acid (LA), an essential long-chain unsaturated FA incorporated by animal from diet is the most abundant FA in follicular fluid (5). It is involved in oocyte maturation and presumably in oocyte competence in development to blastocyst stage. Fatty acids may affect the maturation of oocytes by altering the structure of lipids in oocytes or through changes in type and concentrations of PGs or other metabolites present in oocyte follicular fluid surrounding (10-12). The addition of conjugated isomer of linoleic acid (CLA), the 10t, 12c CLA, to serum-containing IVC media was first described by Pereira *et al* who observed that culturing bovine embryos in 10t, 12c CLA-supplemented medium had no effect on embryonic development to blastocyst stage but increased the embryos ability to maintain their integrity and to re-expand after cryopreservation (13).

It has been previously reported that treatment of bovine cumulus oocyte complexes (COCs) with physiological concentrations of CLA affects the molecular mechanisms that control oocyte nuclear maturation, leading to decreased proportion of oocytes reaching metaphase of second meiotic division (MII) stage at 24 hr of culture, and inhibits subsequent early embryo development (14). Previous studies reported that LA has detrimental effects on oocyte and embryo development, by alteration in GPx and SOD mRNA expression and reproductive hormones (15, 16). The supplementation of media with cis and trans-9, -11 and -10, 12- CLA during IVM and IVC increased the rate of embryonic survival after warming and was related to alter in phospholipids composition of these embryos, which increased the level of unsaturation in blastocysts’ cell membranes and consequently would increase their fluidity (17). 

Fouladi-Nashta *et al* proposed that embryo incorporation of CLA t10, c12 during culture increased membrane fluidity thus conferring embryos with greater resistance to apoptosis (4). Apoptosis is a well-known cell death mechanism, as is necrosis, and apoptosis is regulated by several genes and molecules that play a significant role in initiation of this process. Expression of Bcl-2 (B-cell lymphoma 2) and Bax (Bcl-2- associated X) proteins in oocytes along with embryos of different quality and stages has been assessed by western-blotting analysis by Yang and Rajamahendran (18). They observed the high expression of Bcl-2 in good quality oocytes and embryos, while it was hardly detectable in denuded oocytes. On the contrary, expression of Bax was found in all types of oocytes and highest level was shown in denuded oocytes. Therefore, Yang and Rajamahendran concluded that ratio of Bcl-2 to Bax may be used to predict the tendency of oocytes towards either survival or apoptosis. In many studies estimating the apoptotic genes expression as a quality marker for oocytes and embryos, assessment of apoptotic genes expression is more frequently performed at RNA level than at protein level (18). These investigators also demonstrated that the ratio of expression of Bcl-2 to Bax is critical determinant of either cell survival or death. Also no literature has been found on presence and function of Bcl-2 and Bax gene in ovine embryos at different concentration of LA. 

Therefore present study conducted to investigate effect of LA addition to in vitro maturation culture on ovine embryo development and apoptotic related gene expression.

## Materials and methods

**Reagents and media**

This experimental study was carried out on 450 ovine cumulus-oocyte complexes (COCs) with homogenous ooplasm and more than two compact layers of cumulus cells from September 2013 to September 2014 at Tabriz University. The study protocol was approved by (Tabriz University) Research Council. 

Chemicals were purchased from Sigma (Sigma- Aldrich Corp., St. Louis, MO, USA). All of the reagents were tested for cell or embryonic culturing. At the moment of use, the LA stock solution was diluted in maturation medium (TCM-199) to a final concentration of 100 mM. For in vitro maturation the COCs were randomly allocated to four treatment groups with four replicate in each group which consist approximately 25 COCs for a period of 24 hr. Treatment groups were as follow: control maturation media, 0 µM LA; 50 µM LA; 100 µM LA and 200 µM LA. In experiment 1, cumulus cell expansion was recorded and oocytes were denuded, fixed, and stained to assess the stage of nuclear maturation. In experiment 2, treated COCs were in vitro fertilized and cultured for 6 days. Cleavage and blastocyst rates were recorded on days 1 and 6 respectively. In experiment 3, total RNA was extracted from embryo and reverse transcribed, and mRNA expression of Bcl2 and Bax genes was determined using Real Time PCR method.

**Collection of oocytes**

Ovine ovaries were obtained (with ethical respect) from a local abattoir and transported to laboratory (University of Tabriz) in PBS supplemented with penicillin (59 mg/L) and streptomycin (100 mg/L) at 30-35^o^C within 2 hr after slaughter. At the laboratory, 2-6-mm follicles were aspirated to obtain the COCs. Afterward, the COCs were transferred into a 35-mm Petri dish and washed twice before moving to maturation medium (19).

**In vitro maturation (IVM)**

COCs with two layers or more of compact cumulus cells were selected for maturation. After washing, COCs were transferred in 200 µl micro drops of TCM-199 supplemented with 10% FBS, 1 µg/mL 17 β-estradiol, 0.5 µg/mL follicle stimulating hormone, 0.5 µg/mL luteinizing hormone, 100 IU/mL penicillin and 100 µg/mL streptomycin under mineral oil in maximum humidified with 5% CO_2_ at 38.5^o^C for 24 hr (15, 25).

**Assessment of cumulus cell expansion**

The degree of cumulus expansion was assessed under a stereomicroscope after 24 hr of maturation subjectively as not expanded, partially expanded (outer layers of cells were loosened), or fully expanded (all cumulus cells were loosened) (19).

**Oocyte staining and determination of stage of nuclear maturation**

Oocyte nuclear stage in meiosis was determined after aceto-orcein staining (19). Briefly, the oocytes were denuded by gentle pipetting and placed on a clean glass slide and overlaid with a square cover slip that was held up by four droplets of a vaseline-paraffin mixture (40:1). Afterward, they were fixed for at least 24 hr in glacial acetic acid (GAA) in methanol fixative solution (1:3). Thereafter, the oocytes were stained for 2 min with 1% orcein in a 45% GAA mixture before washing with a mixture of distilled water, glycerol, and GAA (3:1:1). Finally, the nuclear maturation was recorded under a phase contrast microscope (Labomed TCM 400).

**In vitro fertilization**

In vitro fertilization was performed according to standard procedures previously described with some modification (20). Following maturation, cumulus cells were partly removed by gentle pipetting before oocytes were placed in 100 µl fertilization microdrops of synthetic oviduct fluid (SOF) containing 4 IU/ml heparin, PHE (20 µM penicillamine, 10 µM hypotaurine, 1 µM epinephrine) and 2% (v/v) estrous ovine serum overlaid with mineral oil. The frozen-thawed semen was thawed in water bath at 37^o^C. Motile spermatozoa were obtained for fertilization by using swim-up method. 

Briefly, motile sperms were selected by swim-up method for 45 min in calcium-free medium followed by centrifugation at 300× gr at 20^o^C and resuspension of pellet in fertilization medium (Tyrode’s albumin- lactate-pyruvate media supplemented with 0.6% [wt/vol] fatty acid-free BSA,1 mg/mL heparin, 50 ng/mL epinephrine, and 50 ng/mL hypotaurine). After pellet was resuspended, sperm concentration was determined by using hemocytometer. Sperm were then diluted in HEPES-Synthetic oviduct fluid (HSOF), which would produce a 1×10^6^ spermatozoa/ml at final concentration. COCs (25 per 200-µL droplet), and spermatozoa were co-incubated for 18 hr under the same conditions used for IVM. The day of fertilization was defined as Day 0 (16).

**In vitro embryo culture**

18 hr after insemination, presumptive zygotes were transferred into 25 µL droplets of SOF supplemented with BME and MEM amino acids and bovine serum albumin (6 mg mL-1 BSA) until the stage of 2-8 cells. After assessing cleavage using a stereo microscope (Olympus SZ60), embryo development proceeded until the blastocyst stage in amino acids and BSA supplemented SOF plus 10% fetal calf serum (SOF serum). Embryo culture was performed at 38.5^o^C in a humidified atmosphere with 5% O_2_, 5% CO_2_ and 90% N_2_. Cleavage rate was calculated as number of cleaved embryos per inseminated oocytes number. D6 embryo developmental rates were calculated as number of D6 blastocysts per cleaved embryos number , respectively. Embryos were placed in dry Eppendorf tubes and stored at -80^o^C until RNA extraction. 

**RNA extraction, cDNA synthesis and relative quantification by real-time PCR**

Total RNA was extracted from blastocytes. Transcript abundance of Bax, Bcl2 and GPDH (housekeeping gene) were analyzed using qPCR. Simultenus RNA extraction and cDNA synthesis performed according to previous published papers (21). Briefly, three pools of biological replicates, each containing five embryos at blastocyst stages were transferred to Eppendorf tube and used for production of cDNA in follow added 1.25 µL Taq Polymerase, 20.75 µL Master Mix (Takara), and 2 µL specific primers to each 2µL cDNA for PCR mixture. Real-time PCR reactions were carried out in total volume of 13 µL according to manuals for DNA Master SYBR Green I mix (Roche Applied Sciences). Primers used information for real-time PCR is listed (Table IV). Samples were analyzed in duplicate, and duplicate average values was used for quantification. Data were normalized to GAPDH and 2-∆∆Ct methodology was used for relative quantification.

**Statistical analysis**

All experiments were repeated at least three times. Developmental rates from zygote to different stages, cell numbers were subjected to one-way ANOVA using a SPSS (Version 21; SPSS Inc., Chicago, IL, USA). Differences among treatment means were analyzed using Tukey test. Differences with p<0.05 were considered significantly different.

## Results

**Cumulus cell expansion**

Highest concentration (200 µM) of LA significantly decreased the fully expanded cumulus cells rate 24 hr after IVM compared with control (p<0.05, Table I).

**Determination of the stage of nuclear maturation**

Results from oocyte maturation did not show any difference on oocyte degeneration, germinal vesicle (GV), germinal vesicle breakdown (GVBD), MI (metaphase I), AI (anaphase I), and TI (telophase I) rates at 24 hr of IVM among experimental groups (Table II). On the contrary, significantly lower rates of MII oocytes were found in 200 µM LA group compared with 100 µM LA (90 vs. 97%) (Table II)

**Cleavage rate and embryo development**

Cleavage rates of those zygotes were different among LA concentrations (range 27-52%). LA supplementation had effect on oocytes cleaved percentage to two and four cells at Day 2 post fertilization. The percentage of zygotes developing to blastocysts in 200 µM LA was lower than other group (27.67±0.17 vs. 45.92±0.06, p<0.05). In contrast percentage of zygotes blastocysts developing in 100 µM LA was higher than 50 and 200 groups (Table III). Nevertheless, there was difference among LA concentrations and control groups in blastocyst or hatching rates (Table III).

**Gene expression**

**Figure 1:**
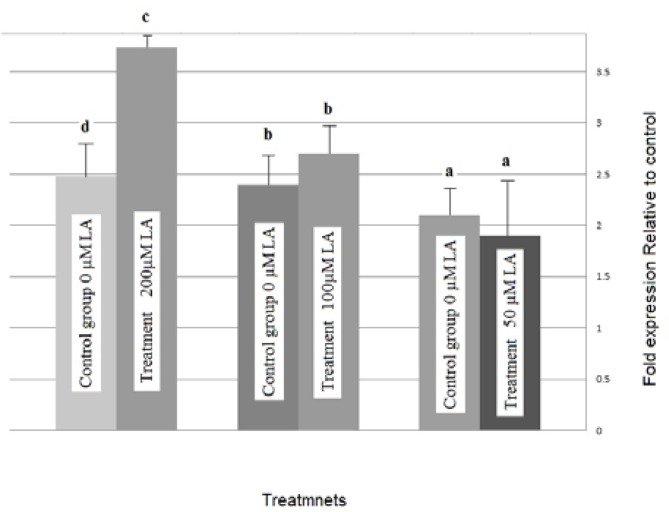
Relative mRNA expression of Bax transcripts of ovine blastocyst derived from LA- treated compared with blastocyst of control oocytes. A: Linoleic acide, Bars with different letters represent groups that were different from control group (p˂0.05

**Figure 2:**
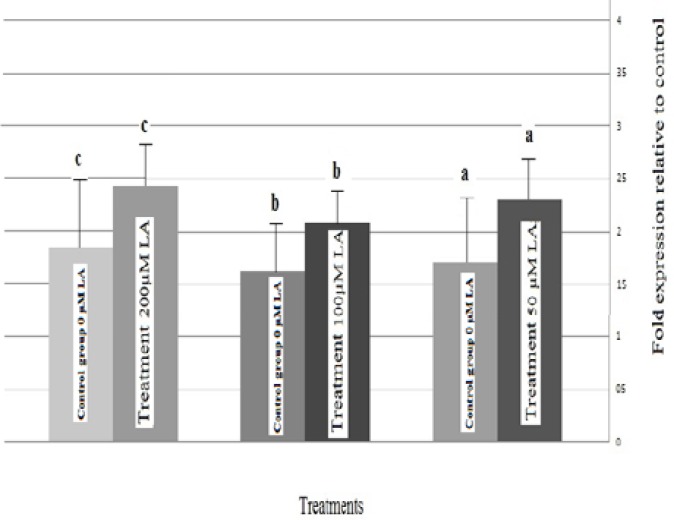
Relative mRNA expression of Bcl2 transcripts of ovine blastocyst derived from LA- treated compared with blastocyst of control oocytes. A: Linoleic acide, Bars with different letters represent groups that were different from control group (p˂0.05).

Supplementation of oocyte maturation media with 200µ MLA significantly increased Bax mRNA expression (p=0.05). However, treatment with 100µM LA-supplemented media decreased Bax mRNA (Figure 1). We did not observe changes in relative abundance of Bcl2 gene expression in ovine embryo after IVM of ovine oocytes with high or low concentration LA (Figure 2).

**Table I:** Effect of LA concentrations (50, 100, and 200µM) added to IVM media on cumulus cell expansion of ovine oocyte 24 hr after IVM.

**Treatments **	**Total number of COCs**	**Fully expanded COCs **	**Partially expanded COCs **	**Not expanded COCs **
Control	90	75 (83.33)^a^	10 (11)	10 (11)
LA 50µM	120	44 (36.66)^b^	39 (32.5)	17 (14.16)
LA 100µM	120	46 (38.33)^b^	35 (29.16)	19 (15.83)
LA 200µM	120	39 (32.5)^ab^	30 (25)	31 (25.83)

Data are presented as n (%).

Letters a, b indicate significant differences (p<0.05) among experimental groups for fully expanded cumulus oocytes.

COCs: cumulus oocyte complexes

LA: linoleic acid.

**Table II:** Comparison of different concentration of LA (50, 100, and 200μM) in IVM media on nuclear maturation stages of ovine oocytes 24 hr after IVM

**Treatments**	**Total oocyte**	**Gv**	**GVBD**	**MI**	**AI**	**TI**	**MII**	**Degenerated oocytes**
Control	90	4 (4.44)	0	0	0	0	83 (92.22)^a^	3 (3.34)
LA 50µM	120	2 (1.66)	1 (0.8)	0	0	0	112 (93.38)^a^	5 (4.16)
LA 100µ	120	3 (2.5)	0	0	0	0	115 (95.84) ^ab^	2 (1.66)
LA 200µM	120	3 (2.5)	2 (1.66)	0	0	0	108 (90)^b^	7 (5.84)

Within the columns, the values did differ significantly (p<0.05) according to Tukey’s test. Different superscript letters indicate significant differences among experimental groups (p<0.05). Data are presented as n (%).

LA: linoleic acid

GV: germinal vesicle

GVBD: germinal vesicle breakdown.

MI: metaphase of the first meiotic division; anaphase of the first meiotic division

MII: metaphase of the second meiotic division.

TI: telophase of the first meiotic division

**Table III:** Effects of the timing of LA supplementation during the in vitro production ovine embryos on the cleavage rates and the embryonic development to the blastocyst stage

**Experiments **	**Total oocyte**	**Cleavage **	**Two cell**	**Four cell**	**Blastocysts **
Control	100	63.33±0.14^a^	18.33±0.04^a^	25±0.86^a^	20±0.28^a^
50µM	110	52.33±0.06^a^	12.67±0.08^a^	26±0.08^a^	13.33±0.13^b^
100µM	110	40.33±0.07^a^	21±0.3^a^	50±0.5^b^	14±0.1^b^
200µM	130	27.67±0.17^b^	19±0.1^a^	9.33±0.05^a^	12.67±0.01^b^

The data are presented as the mean±SD%.

Within the columns, the values did differ significantly (p<0.05) according to Tukey’s test.

Different letters (a, b) indicate significant differences among experimental groups (p<0.05).

**Table IV:** Details of primers used for real-time PCR quantitative analysis

**Gene name **	**Primer sequences**	**Annealing temperature (c)**	**Size**	**Gen accession number**
Bax–F	F:5′-TGGAGATGAATTGGACAGTAAC-3′	61	174bp	XM_004015363.1
Bax-R	R:5′-GCCTTGAGCACCAGTTTG-3′			
Bcl2-F	F:5′- CAGGAGAAATCAAACAGGG-3	59	171bp	XM004020687
Bcl2-R	R:5′-GTGTGTGGAGAGCGTCAAC-3′			
GAPDH-F	F:5′-AGTGTCGCTGTTGAAGTCG-3′	60	121bp	NM_001190390.1
GAPDH-R	R:5′-GAAACCTGCCAAGTATGATG-3′			

PCR: polymerase chain reaction

## Discussion

PUFAs constitute major portion of fatty acid content of bovine follicular fluid. Among them, LA is dominant one. It contributes about one-third of total fatty acid composition, and its concentration varies with size of follicle; large follicles have significantly lower concentrations than small follicles (5). Diets high in n-6 have been associated with lower pregnancy rate; however, not all results are consistent. For example, pregnancy rates were higher when Bosindicus Nelore beef cows received a supplement containing short-chain n-6 LA or when Holstein dairy cows received supplement containing calcium salts of n-6 compared with saturated fat (22-24). 

Our results revealed that LA at high concentration (200 µM) reduced the number of COCs with fully expanded cumulus cells. This was in agreement with published results on maturation of bovine oocytes using this high LA concentration (25). There was also a reduction in rate of oocytes reaching MII stage. These results confirmed that high concentrations of ALA could be harmful for prepubertal sheep oocytes too. Thus, it seems that 200 µM is an excessive concentration compared with physiological levels measured in sheep and bovine (15). This shows that the mechanism by which high concentration of LA inhibits cumulus cell expansion is similar from that involved in oocyte nuclear maturation. It seems that these two processes are linked together. 

In fact, cumulus cell expansion has been shown to be prerequisite for nuclear maturation and development of ovine COCs (24). This finding is apposite with the study of Khalil *et al* who showed that cumulus cell expansion not prerequisite for nuclear maturation (16). Also Marei *et al* observed no effects on oocyte maturation and cumulus expansion of bovine oocytes incubated in presence of LA at 50 µM (14). In summary, inhibitory effects of high concentration LA on cumulus cell expansion and oocyte nuclear maturation takes place through different routes, and oocyte nuclear maturation may be affected by oxidative stress induced by LA.

Oocyte maturation is fundamental process in which oocyte arrested at prophase I (since fetal life) ultimately resumes meiosis and prepares for subsequent fertilization. Under our experimental conditions, at least up to a concentration of 100 µM, LA had no apparent negative effects on bovine oocyte maturation competence. Results of the present study did not confirm previous findings that 100 µM concentration of LA in IVM medium inhibited maturation progression, manifested by high percentage of oocytes arrested at GVBD stage and a concomitant decrease in percentage of oocytes that completed second meiotic division metaphase (16). 

Carro *et al* observed that LA added to defined maturation medium at concentrations that did not alter the nuclear status of bovine oocytes matured in vitro (9 and 43 µM) improved their quality by increasing content of neutral lipids stored in lipid droplets (26). Leão *et al* reported that presence of conjugated LA isomer (trans-10 cis-12 conjugated linoleic acid, CLA) in IVM medium at same concentration (100 µM) did not alter oocyte maturation or embryo production rates (17). Khalil *et al* showed that supplementation with n-6 may be detrimental to oocyte maturation, as high concentrations of n-6 in follicular fluid surrounding immature oocyte and oocyte itself (5, 16, 27).

This discrepancy could be partly because of different experimental conditions, concentration and use of different LA sources. LA has also been shown to stimulate production of ROS in other cell types and oxidative stress (induced by H_2_O_2_ treatment) during maturation results in disruption of meiotic spindles in mouse oocytes (28). Our results showed that 200 µM concentration is almost lethal for ovine embryo production leading to lower blastocyst rate (p<0.05, Table II). This is in agreement with the study of Medina *et al* who reported that supplementation with either CLA isomer did not improve embryo production (29). It is possible that in absence of free radical scavenger, this PUFA supplementation to embryo culture media may exert a detrimental effect (16, 30). 

Stinshoff *et al* also demonstrated a decrease in cattle blastocysts developmental rate in CLA groups either with 50 or 100-µM doses supplementation (31). We observed higher cleavage rate and embryo development resulting from oocytes matured in TCM supplemented with LA-100. This is in agreement with the study of Ghaffarilaleh *et al* who reported that, addition of ALA to IVM media of prepubertal sheep oocytes could improve their embryo development (15). In cattle, the addition of 50 µM of ALA to IVM has improved oocyte nuclear maturation and embryo development (32). This effect is mediated both directly through Mitogen-Activated Protein Kinase pathway and indirectly through PGE2 synthesis and changing mitochondrial distribution and activity (14, 32). This is in contrast with a report from Wonnacott *et al* who did not find any improvement in cleavage and blastocyst rates when adding omega-3 or omega-6 HDL to IVM media of ovine oocytes (7). Nonetheless, these effects also seem to depend on the type of LA used.

Low concentration of LA added to IVM medium in the present study did not affect the level of Bax gene transcripts in blastocysts produced. However, we observed a trend for up-regulation of Bax gene in embryos resulting from oocytes matured in culture medium supplemented with 200µM LA. Whereas in contrary study by Knijn *et al*, mRNA expression levels for Bax, were not affected by production method (33). Also in this study we did not observe changes in relative abundance of Bcl-2 gene expression in ovine embryo after IVM of ovine oocytes with high or low concentration LA. 

Thus Bcl-2 is not reliable and no practicable alternative is available at the moment, making it impossible to predict the survival or apoptotic chance of cell based on Bcl-2/ Bax ratios. It is noteworthy that embryo culture conditions can also affect gene expression in blastocysts and mask possible effects of IVM systems on embryos.

## Conclusion

In conclusion, the data obtained in the present study suggest that high concentration of LA supplementation of culture media significantly reduced the COCs exhibiting full cumulus cell expansion percentage, oocytes reaching metaphase II stage, and lowered the blastocyst rate compared with controls. However, based on current findings low concentration of LA used for maturation had no deleterious effect on subsequent embryonic development compared to high concentration of LA. The relative expression of Bcl-2 and Bax in embryos seems to be associated with LA concentration.

## Note

***This article extracted from D.V.M. thesis. (Ebrahim Amini)***

## References

[1] Santos JE, Bilby TR, Thatcher WW, Staples CR, Silvestre FT (2008). Long chain fatty acids of diet as factors influencing reproduction in cattle. Reprod Domest Anim.

[2] Spector AA (1999). Essentiality of fatty acids. Lipids.

[3] SampathH, Ntambi JM (2005). Polyunsaturated fatty acid regulation of genes of lipid metabolism. Ann Rev Nutr.

[4] Fouladi-Nashta AA, Wonnacott KE, Gutierrez CG, Gong JG, Sinclair KD, Garnsworthy PC (2009). Oocyte quality in lactating dairy cows fed on high levels of n-3 and n-6 fatty acids. Reproduction.

[5] Homa S, Brown C (1992). Changes in linoleic acid during follicular development and inhibition of spontaneous breakdown of germinal vesicles in cumulus-free bovine oocytes. J Reprod Fertil.

[6] Sturmey R, Reis A, Leese H, McEvoy T (2009). Role of fatty acids in energy provision during oocyte maturation and early embryo development. Reprod Domest Anim.

[7] WonnacottKE, Kwong WY, Hughes J, Salter AM, Lea RG, Garnsworthy PC (2010). Dietary omega-3 and -6 polyunsaturated fatty acids affect the composition and development of ovinegranulosa cells, oocytes and embryos. Reproduction.

[8] Hughes J, Kwong WY, Li D, Salter AM, Lea RG, Sinclair KD (2011). Effects of omega-3 and -6 polyunsaturated fatty acids on ovine follicular cell steroidogenesis, embryo development and molecular markers of fatty acid metabolism. Reproduction.

[9] AardemaH, Vos PL, Lolicato F, Roelen BA, Knijn HM, Vaandrager AB (2011). Oleic acid prevents detrimental effects of saturated fatty acids on bovine oocyte developmental competence. Biol Reprod.

[10] Gulliver CE, Friend MA, King BJ, Clayton EH (2012). The role of omega-3 polyunsaturated fatty acids in reproduction of ovine and cattle. Anim Reprod Sci.

[11] Fouladi-Nashta AA, Gutierrez CG, Gong JG, Garnsworthy PC, Webb R (2007). Impact of dietary fatty acids on oocyte quality and development in lactating dairy cows. Biol Reprod.

[12] Bender K, Walsh S, Evans AC, Fair T, Brennan L (2010). Metabolite concentrations in follicularfluid may explain differences in fertility between heifers and lactating cows. Reproduction.

[13] Pereira RM, Baptista MC, Vasques MI, Horta AE, Portugal PV, Bessa RJ (2007). Cryosurvival of bovine blastocysts is enhanced by culture with trans-10 cis-12 conjugated linoleic acid (10t, 12c CLA). Anim Reprod Sci.

[14] Marei WF, Wathes DC, Fouladi-Nashta AA (2010). Impact of linoleic acid on bovine oocyte maturation and embryo development. Reproduction.

[15] Ghaffarilaleh V, Fouladi-Nashta AA, Paramio MT (2014). Effect of α-linolenic acid on oocyte maturation and embryo development of prepubertal sheep oocytes. Theriogenology.

[16] Khalil WA, Marei WFA, Khalid M (2013). Protective effects of antioxidants on linoleic acid-treated bovine oocytes during maturation and subsequent embryo development. Theriogenology.

[17] Leão BCS, Rocha-Frigoni NAS, Cabral EC, Coelho MB, Ferreira CR, Eberlin MN (2015). Improved embryonic cryosurvival observed after in vitrosupplementation with conjugated linoleic acid is related tochanges in the membrane lipid profile Theriogenology.

[18] Yang MY, Rajamahendran R (2002). Expression of Bcl-2 and Bax proteins in relation to quality of bovine oocytes and embryos produced in vitro. Anim Reprod Sci.

[19] Marei WF, Wathes DC, Fouladi-Nashta AA (2009). The effect of linolenic acid on bovine oocyte maturation and development. Biol Reprod.

[20] Cognié Y, Baril G, Poulin N, Mermillod P (2003). Current status of embryo technologies in ovine and goat. Theriogenology.

[21] Dehghani-Mohammadabadi M, Salehi M, Farifteh F, Nematollahi S, Arefian E, Hajjarizadeh A (2014). Melatonin modulates the expression of BCL-xl and improve the development of vitrified embryos obtained by IVF in mice. J Assist Reprod Genet.

[22] Lopes CN, Scarpa AB, Cappellozza BI, Cooke RF, Vasconcelos JLM (2009). Effects of rumen-protected polyunsaturated fatty acid supplementation on reproductive performance of Bosindicus beef cows. J Anim Sci.

[23] Juchem SO, Cerri RL, Villasenor M, Galvao KN, Bruno RG, Rutigliano HM (2010). Supplementation with calcium salts of linoleic and transoctadecenoic acids improves fertility of lactating dairy cows. Reprod Domest Anim.

[24] Ambrose DJ, Kastelic JP, Corbett R, Pitney PA, Petit HV, Small JA (2006). Lower pregnancy losses in lactating dairy cows fed a diet enriched in alpha-linolenic acid. J Dairy Sic.

[25] Marei WF, Wathes DC, Fouladi-Nashta AA (2012). Differential effects of linoleic and alpha-linolenic fatty acids on spatial and temporal mitochondrial distribution and activity in bovine oocytes. Reprod Fertil Dev.

[26] Carro M, Buschiazzo J, Ríos GL, Oresti GM, Alberio RH (2013). Linoleic acid stimulates neutral lipid accumulation in lipid drop lets of maturing bovine oocytes. Theriogenology.

[27] Kim JY, Kinoshita M, OhnishiM, FukuiY (2001). Lipid and fatty acid analysis of fresh and frozen-thawed immature and in vitro matured bovine oocytes. Reproduction.

[28] Zhang X, Wu XQ, Lu S, Guo YL, Ma X (2006). Deficit of mitochondria derived ATP during oxidative stress impairs mouse MII oocyte spindles. Cell Res.

[29] Absalón-Medina VA, Bedford-Guaus SJ, Gilbert RO, Siqueira LC, Esposito G, Schneider A, Cheong SH (2014). The effects of conjugated linoleic acid isomers cis-9, trans-11 and trans-10,cis-12 on in vitro bovine embryo production and cryopreservation. J Dairy Sci.

[30] Reis A, Rooke JA, McCallum GJ, Staines ME, Ewen M, Lomax MA (2003). Consequences of exposure to serum, with or without vitamin E supplementation, in terms of the fatty acid content and viability of bovine blastocysts produced in vitro. Reprod Fertil Dev.

[31] Stinshoff H, Wilkening S, Hanstedt A, Bollwein H, Wrenzycki C (2014). Dimethylsulfoxide and conjugated linoleic acids affect bovine embryo developmentin vitro. Reprod Fertil Dev.

[32] Marei WF, Wathes DC, Fouladi-Nashta AA (2009). The effect of linolenic acid on bovine oocyte maturation and development. Biol Reprod.

[33] Knijn HM, Wrenzycki C, Hendriksen PJM, VosPLAM, Zeinstra EC, van der Weijden GC (2005). In vitro and in vivo culture effects on mRNA expression of genes involved in metabolism and apoptosis in bovine embryos. Reprod Fertil Dev.

